# Physiology can contribute to better understanding, management, and conservation of coral reef fishes

**DOI:** 10.1093/conphys/cox005

**Published:** 2017-02-23

**Authors:** Björn Illing, Jodie L. Rummer

**Affiliations:** 1 Australian Research Council Centre of Excellence for Coral Reef Studies, James Cook University, Townsville, QLD 4811, Australia; 2 Institute of Hydrobiology and Fisheries Science, University of Hamburg, Hamburg D-22767, Germany

**Keywords:** Adaptive management, conservation, coral reef, elasmobranch, teleost, tolerance

## Abstract

Coral reef fishes, like many other marine organisms, are affected by anthropogenic stressors such as fishing and pollution and, owing to climate change, are experiencing increasing water temperatures and ocean acidification. Against the backdrop of these various stressors, a mechanistic understanding of processes governing individual organismal performance is the first step for identifying drivers of coral reef fish population dynamics. In fact, physiological measurements can help to reveal potential cause-and-effect relationships and enable physiologists to advise conservation management by upscaling results from cellular and individual organismal levels to population levels. Here, we highlight studies that include physiological measurements of coral reef fishes and those that give advice for their conservation. A literature search using combined physiological, conservation and coral reef fish key words resulted in ~1900 studies, of which only 99 matched predefined requirements. We observed that, over the last 20 years, the combination of physiological and conservation aspects in studies on coral reef fishes has received increased attention. Most of the selected studies made their physiological observations at the whole organism level and used their findings to give conservation advice on population dynamics, habitat use or the potential effects of climate change. The precision of the recommendations differed greatly and, not surprisingly, was least concrete when studies examined the effects of projected climate change scenarios. Although more and more physiological studies on coral reef fishes include conservation aspects, there is still a lack of concrete advice for conservation managers, with only very few published examples of physiological findings leading to improved management practices. We conclude with a call to action to foster better knowledge exchange between natural scientists and conservation managers to translate physiological findings more effectively in order to obtain evidence-based and adaptive management strategies for the conservation of coral reef fishes.

## Teaser

Here, we provide an overview of how physiological research supports evidence-based conservation management of coral reef fishes. In recent years, this connection has improved, but the implementation of management based on physiological findings still needs better advocacy to result in effective conservation of coral reef fish populations and ecosystems.

## Introduction

Coral reef ecosystems provide millions of people with numerous services, ranging from coastal protection to providing habitat for fish species that support both thriving tourism and fisheries sectors (Hicks, 2011; Teh *et al*., 2013). In addition to their socio-economic value, coral reefs represent habitats with the highest biodiversity in the marine realm and accommodate ~5000 fish species (Bellwood *et al*., 2012). This means that coral reef and reef-associated fish species represent about one-third of all brackish and marine fish species worldwide, considering the 28 000–32 200 globally known fish species (Nelson, 2006; FishBase, 2016). While many coral reef fishes populated reef habitats 65–50 million years ago, and since then have experienced and successfully adapted to variations in environmental conditions, others diversified more recently over the geological time scale (23–3 million years ago) and may have only experienced relatively stable environmental conditions (see references in Rummer and Munday, 2017). Today, coral reef fishes are facing anthropogenic climate change and therefore changes in their environmental conditions at an unprecedented rate. In contrast to the continuously but slowly changing conditions of the past, climate change projections of increased water temperatures, reduced ocean pH and reductions in dissolved oxygen will catapult coral reef fishes and other marine organisms currently populating low-latitude regions into more stressful and potentially hazardous conditions before the end of this century (Rummer *et al*., 2014; Rummer and Munday, 2017). Elevated water temperatures have already resulted in successive mass bleaching events on coral reefs worldwide, with transient warming events predicted to be more severe and frequent in coming years. In 2016, for example, 93% of the Great Barrier Reef in Australia bleached owing to climate change-driven warming waters, which resulted in unprecedented levels of coral mortality (Ainsworth *et al*., 2016). Moreover, the global increase in human populations and concentrations near coastal areas will exacerbate the direct pressure on coral reef fish populations through fishing pressure (Roberts, 1995; Allgeier *et al*., 2016), recreational activities (e.g. angling, diving, boat traffic; Mandelman and Skomal, 2009; Gil *et al*., 2015; Berthe and Lecchini, 2016) and coastal development, with the often accompanying increases in agricultural and industrial runoff and pollutants (D'Angelo and Wiedenmann, 2014; Kroon *et al*., 2014, 2016). In order to prevent a loss of biodiversity and to foster a sustainable use of the resources of coral reef ecosystems, there is a strong need for research that identifies and quantifies species, populations and habitats that are most at risk and programmes to translate these findings into implementing management and policy over both short- and long-term time scales.

**Figure 1:**
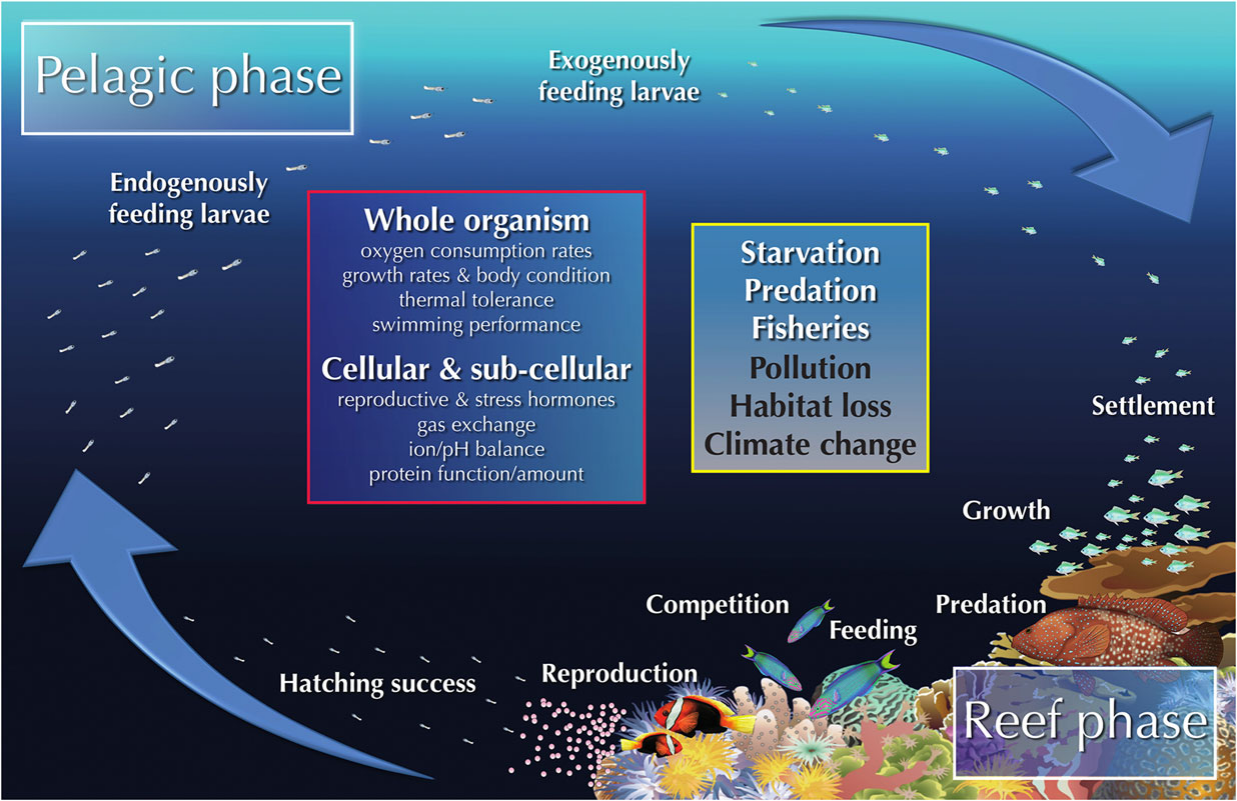
Schematic life cycle of a model coral reef fish (e.g. Pomacentridae). The yellow box indicates direct (white text) and indirect (black text) sources of mortality. The red box represents experimental approaches that have been suggested or implemented in coral reef fish conservation management (see main text for further details). Fish images are courtesy of Erin Walsh. Coral images are courtesy of the Integration and Application Network, University of Maryland Center for Environmental Science (ian.umces.edu/symbols/).

Indeed, the importance of coral reef fish conservation biology has perhaps never been more evident, and studies over the past two decades have resulted in the development of an extensive knowledge base covering key processes that affect different life stages of coral reef fishes (Hixon, 2011). During their life cycle, coral reef fishes face stage-specific risks (Fig. 1), but like most other fish species, their eggs and larvae are the most vulnerable life stages and are strongly affected by predation (Hixon, 1991). Hence, most coral reef fish species shift habitats during this stage and possess a pelagic phase post-hatching to optimize growth and reduce predation pressure (Grol *et al*., 2014; Fig. 1). The reef phase starts when late larval stage fishes undergo metamorphosis. During this time, larvae settle onto the reef, where predation-induced mortality rates can exceed 70–90% after a single day (Doherty *et al*., 2004; Fig. 1). Surviving this decisive window of vulnerability, which poses a bottleneck at the population level, the juvenile fishes feed and grow on the reef, transition into adults and close the life cycle by reaching the reproductive stage (Fig. 1). During both pelagic and reef phases, various factors affect growth, condition and survival of coral reef fishes, including inter- and intra-specific competition for food (Bonin *et al*., 2015), habitat loss and pollution (Wilson *et al*., 2010b; Wenger *et al*., 2015), and fish can also be subject to predation and fisheries pressure (Graham *et al*., 2007; Boaden and Kingsford, 2015; Fig. 1). Climate change will add to these aforementioned factors, with consequences for survival at the species and population levels as well as the health of coral reef ecosystems (Rummer and Munday, 2017). Therefore, the key to ensuring a sustainable use and management of coral reef fishes will be a holistic understanding of how physiological processes respond to anthropogenic stress and environmental change (Wilson *et al*., 2010a; Doney *et al*., 2012; Cooke *et al*., 2013b).

Various physiological measurements have been used to investigate coral reef fish performance at the whole-organism level but also at cellular and sub-cellular levels (see Fig. 1 for details) so that ecosystem-level predictions can be made. In fact, a questionnaire from Wilson *et al*. (2010a) to scientists working on reef fish topics revealed that, although only relatively few researchers were working on coral reef fish physiology at the time, the use of physiological methods was considered by all of the polled scientists to have the highest priority for future work on coral reef fishes, because findings had important implications for resilience and management. More specifically, including physiological measurements can lead to higher precision in forecasting individual and species’ responses and reveal underlying, mechanistic cause-and-effect relationships (see references in Coristine *et al*., 2014). Findings can, in turn, be used to provide decision-makers on legislative levels, local conservation managers and stakeholders with more in-depth explanations of observed changes in population declines or habitat shifts (Wikelski and Cooke, 2006; Cooke *et al*., 2013b; Coristine *et al*., 2014). Besides gaining a mechanistic understanding, Somero *et al*. (2016) describe the specific benefits of physiological studies for management and policy, with an increased confidence in modelled scenarios (including estimates of organismal functional responses in projected future conditions), the determination of threshold values and development of indicators that could induce regulatory responses when needed. Thus, considering the admitted potential and implied benefits of using physiological studies for management of coral reef fishes, what is the status of coral reef fish physiology research, and have the proposed recommendations so far been well translated? The aim here is first, to analyse studies that combine physiological approaches with management and conservation recommendations for coral reef fish species. Second, several studies will be highlighted that serve as good examples for future use and implementation of physiological findings into management decisions. Moreover, potential as yet untapped will be addressed and perspectives discussed.

## Methods and Results

**Figure 2:**
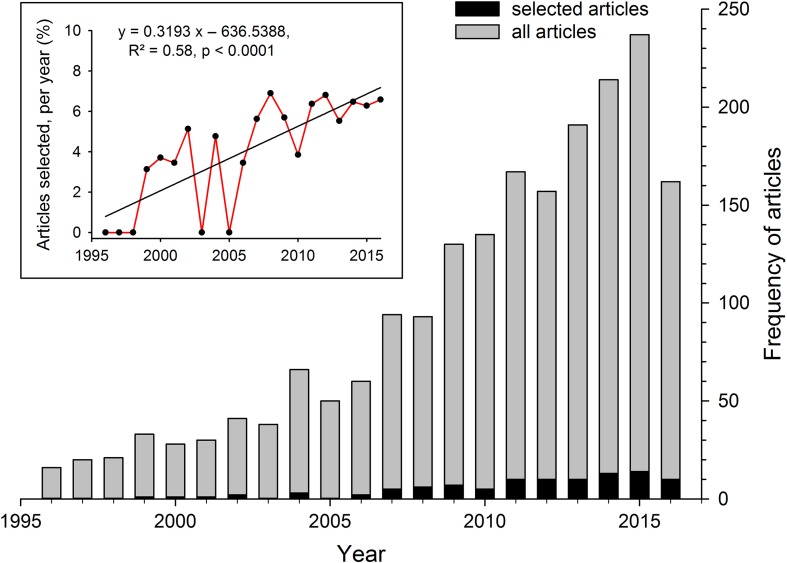
Number of articles (*n* = 1883; grey bars) found during a Thomson Reuter Web of Knowledge literature search (Web of Science Core collection) using coral reef fish-related, physiological and conservation keywords in the topic between 1996 and 2016 (searched on 20 August 2016, hence note the incomplete data set for 2016). The suite of keywords was modified after Lennox and Cooke (2014) and included the search terms (toleran* OR endanger* OR imperil* OR conserv* OR restor* OR manage* OR poli* OR threat* OR decision-making OR protec* OR impact*) AND (toleran* OR physiolog* OR stress* OR energy* OR mechanis* OR threshold OR condition*) AND (coral* OR reef) AND (fish* OR ray OR shark OR teleost* OR elasmo*). The results were screened manually, and relevant publications were found only between 1999 and 2016 (*n* = 99, black bars). The inset graph represents the relative proportion of articles that were manually filtered from the search results for each year (4.0 ± 2.6%; mean ± SD).

To obtain an overview of how well integrated coral reef fish conservation and physiological topics are, a literature search was conducted in Thomson Reuter's Web of Science (on 20 August 2016, using the Web of Science™ Core Collection). Based on two slightly modified suites of conservation and physiology keywords used by Lennox and Cooke (2014), the following keyword complex aimed at finding all primary literature regarding coral reef fish conservation physiology: (toleran* OR endanger* OR imperil* OR conserv* OR restor* OR manage* OR poli* OR threat* OR decision-making OR protec* OR impact*) AND (toleran* OR physiolog* OR stress* OR energy* OR mechanis* OR threshold OR condition*) AND (coral* OR reef) AND (fish* OR ray OR shark OR teleost* OR elasmo*). The obtained results (*n* = 1924 articles) were manually filtered for their relevance, i.e. narrowed down by checking all returned titles, sorting out unsuitable ones and reading the remaining, potentially eligible studies (*n* = 180). Based on their content, fitting studies were manually selected (*n* = 99) and assigned to one or more physiology (*n* = 9) and conservation categories (*n* = 8). With regard to the measurements considered as part of a physiological approach, we used a conservative understanding of physiology, i.e. only studies investigating responses on individual, cellular and sub-cellular levels were used (see description in Fig. 1) but none that, for instance, correlated ecological field observations (such as habitat use, mortality, feeding behaviour or predator–prey interactions). The manually filtered studies, throughout the manuscript termed ‘selected studies’, and their individually assigned categories can be found in the Supplementary material. As no study relevant to conservation physiology of coral reef fishes was found before 1999, only the last 20 years (1996–2016) were selected for investigating possible trends in publications on this topic (Fig. 2). Furthermore, although review articles were used to set the framework of this study and drive the discussion, none was used directly in the analysis.

**Figure 3:**
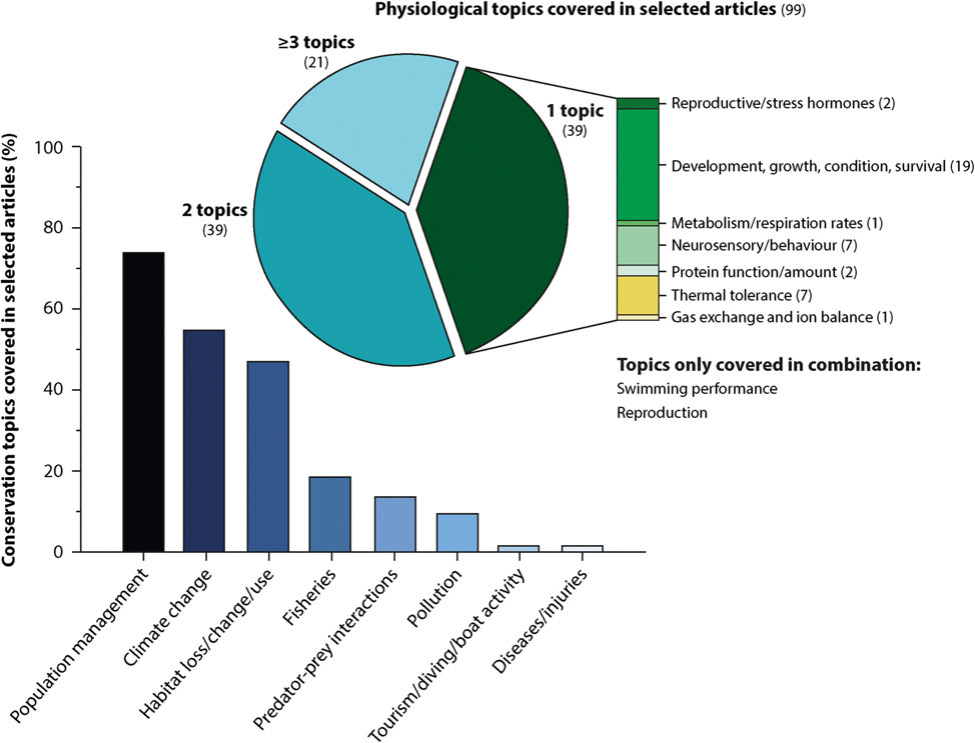
Overview of physiological and conservation topics covered by the selected coral reef fish studies (*n* = 99). The pie chart illustrates how many categories were covered by the individual articles and examines specifically the studies covering only a single aspect to determine which physiological topics were investigated most (number of articles in parentheses). Each bar within the bar plot (eight categories) shows the proportion of studies that covered a certain management or conservation component compared with the total selected articles. Note that most articles covered more than one management or conservation topic, hence the bars collectively add up to exceed 100%.

The number of studies matching the search terms increased over the selected years (note: ongoing 2016 results). To determine whether, in recent years, more studies had matched the search requirements, the relative proportion of articles selected per year was examined in more detail (Fig. 2, inset). From the articles returned by the searched keywords, up to 7% (4.0 ± 2.6%, mean ± SD) were selected per year, which increased significantly over time (linear regression, *R*² = 0.58, *P* < 0.0001). From the 180 studies considered initially, 81 studies (45%) were not chosen because they had no physiological measurement (16%), no conservation advice (13%), or both issues (4%), or they were reviews (10%) or investigated taxonomic groups other than coral reef fishes (2%). From the studies that gave no specific conservation advice, 43% addressed climate change issues (including studies on hypoxia). Regarding the physiological topics, 39% of the selected studies focused on one topic only, and an additional 39% focused on a combination of two physiological topics. The remaining 21% of these studies covered three or more physiological topics in combination (Fig. 3). In the studies that addressed only a single topic, the majority investigated growth, development, condition or survival (*n* = 19), followed by studies investigating thermal tolerance (*n* = 7) or neurosensory/behavioural aspects (*n* = 7). When examining all selected studies, this trend was similar, with growth, development, condition or survival topics being covered by most studies (52%), followed by thermal tolerance (35%), metabolism/respiration rates (29%), neurosensory/behaviour (18%), reproduction (12%), as well as by the other four categories, namely swimming performance, protein function/amount, gas exchange and ion balance, and reproductive/stress hormones. In terms of conservation topics covered by the selected articles, 79% of the studies covered two to three topics, whereas fewer studies addressed a single or four conservation topics (14 and 6%, respectively). The three most covered conservation topics in all selected articles were population management, climate change and habitat loss/change/use, with 74, 55 and 47%, respectively (Fig. 3, bar plot). Topics covered less often included fisheries, predator–prey interactions and pollution (19, 14 and 10%, respectively), or recreational activities and fish-health related topics (2% each).

## Discussion

### Trends in coral reef fish conservation physiology literature

This study presents an overview of the integration status of physiology and conservation topics in coral reef fish studies. At the time of the literature search by Lennox and Cooke (2014), the taxonomic representation of fishes in the conservation physiology literature was the lowest of all vertebrate taxa, but a significantly positive trend in numbers of articles integrating both aspects could be found over recent years. However, the advice given to policy and management seems to differ greatly in terms of practicability and precision. This was mostly caused by the study objectives that were either (i) testing effects of future climate-modulated environmental parameters on coral reef fish physiology, which resulted in rather unspecific and vague advice, if any (see Methods) or (ii) examining tangible and/or more local issues of coral reef fish populations, leading to concrete management recommendations. In the following text, examples from four distinct issues that coral reef fishes are facing are highlighted and summarized.

### Issue I: climate change

The status of the world's biodiversity, and especially the one of threatened species, is currently most affected by overexploitation, agriculture or urban development and far less by climate change (Maxwell *et al*., 2016). However, ‘climate change will become an increasingly dominant problem in the biodiversity crisis’ (Maxwell *et al*., 2016), with ectotherms, such as coral reef fishes, often living close to their thermal tolerance limits, being especially at risk (Rummer *et al*., 2014; Habary *et al*., 2017). In fact, the sensitivity of coral reef fishes to predicted climate change and their physiological responses to living in conditions predicted for the end of the century has been investigated in multifarious studies and extensively reviewed (Munday *et al*., 2008; Rummer and Munday, 2017). Nevertheless, the advice for conservation derived from these physiological studies has so far been largely unprecise, which likely to be because of the uncertain nature of simulation-based thresholds of future marine environments (e.g. 1.5 or 3°C), the large-scale strategies needed for implementation or the simple fact that obvious recommendations for climate protection, such as reducing emissions, have been made repetitively throughout all types of media.

As an example, coral reef researchers experienced, perhaps the first, wake-up call in the 1980s when, on several occasions, warm waters bleached corals worldwide, resulting in widespread mortality and great uncertainty regarding effects on other taxa (e.g. fishes), ecosystem-level responses and recovery rates (Hughes, 2003), a disaster that would become more frequent over subsequent decades. Although coral bleaching, and therefore the stress to the affected coral reef ecosystems, can occur as a result of poor water quality, sedimentation or even uncharacteristically cold waters, most bleaching events on coral reefs worldwide have resulted from warming waters. Elevated sea surface temperatures are attributable to global warming, but natural currents and oceanographic patterns (e.g. El Niño) cause the warm waters to remain on reefs longer than usual (Baker *et al*., 2008). Using the Great Barrier Reef—the largest continuous coral reef system on the planet—as an example, the connection has still not been made to the extent that enough national (e.g. Australia) or global action has been taken, even after the 1997–1998, 2002–2003 and now 2016 bleaching events resulting in mass mortality, with all evidence pointing toward the cause being warming waters attributable to climate change. Is it that researchers have not been communicating thoroughly enough the need to curb human-induced carbon emissions into the atmosphere to slow the warming of the oceans or is it that the solutions are too great to implement? Is there a disconnect between the researchers examining these issues and determining the thresholds for taxa such as the fishes (e.g. +1.5 and +3°C; Donelson *et al*., 2011, 2012; Rummer *et al*., 2014; Habary *et al*., 2017) and those who are making the decisions that, upon implementation, could help to ameliorate these problems or at least reduce the extent or slow the return rate of these events? In this example, coral bleaching has been occurring more and more frequently and to a greater extent over the past couple decades because of 1°C warming (Hughes, 2003; Baker *et al*., 2008), but global initiatives are struggling with 1.5°C, let alone 2°C, targets (Intergovenmental Panel on Climate Change, 2014). In these cases, the gap between researchers and decision-makers seems too great to make a difference, which may be why so many studies lean toward investigating issues with more immediate and tangible outcomes for management.

### Issue II: pollution

The most concrete recommendations to policy-makers and conservation practitioners stem from studies with physiological approaches revealing mechanistic or cause-and-effect relationships with spatially and temporally restricted stressors. One of these stressors can be pollution, and marine habitats are, next to the accumulation of plastic debris, especially challenged by the increasing use of fossil fuel globally. The occasional release of various forms of fossil fuels during production and transport, as well as higher shipping transport and related port expansions, often leads to increased concentrations of suspended sediments and coal dust. Coastal coral reef habitats are prone to these stressors, and coral reef fishes, especially their early life stages, show significantly reduced growth rates and condition, extended larval development, as well as changes in gill structure and gill microbiome when exposed to suspended sediments and coal dust (Wenger *et al*., 2012, 2014; Hess *et al*., 2015; Berry *et al*., 2016; O'Connor *et al*., 2016). All of this information delivers precise threshold-level values for individual organisms to conservation managers and highlights the risk potential for changes at the population and even ecosystem level if no action is taken.

Next to compound-based pollution types, physical pollution, such as boat noise, affects marine organisms and can have fitness consequences for fishes, such as reduced growth and reproduction, or affect their distribution, communication and predator–prey interactions (reviewed by Slabbekoorn *et al*., 2010). Holles *et al*. (2013) were able to show that even local, low-intensity noise has the capacity to disrupt settlement in coral reef fish by scaring their larvae, on top of their natural predator evasion, further away from reef habitats during daytime. Even after successful settlement, there can be considerable consequences for coral reef fish population dynamics, with boat noise, for example, reducing oxygen consumption rates of Ambon damselfish (*Pomacentrus amboinensis*) and doubling its predation risk to another coral reef fish, the dusky dottyback (*Pseudochromis fuscus*; Simpson *et al*., 2016). The advice from both studies for conservation practitioners therefore includes regulating the use of small-scale noise sources (e.g. outboard motorboats) in protected areas and during fisheries management. Overall, the studies highlighted in this section demonstrate the extensive potential for using physiological measurements to inform conservation managers about specific threshold values or concentrations of physical and chemical pollution that trigger negative effects for coral reef fishes.

### Issue III: fishing

Physiological approaches can also help to inform conservation practitioners with very precise advice for recreational fishing activities (Cooke and Schramm, 2007; Cooke *et al*., 2013a). Some coral reef fishes, especially apex predators such as sharks, are popular for trophy and sport fishing, and physiological data can help to examine the stress associated with catch-and-release practices with different gears and how much they contribute to post-release mortality. Several studies have used blood physiology parameters (e.g. pH, partial pressure of CO_2_, lactate, glucose) to investigate physical trauma (hooking injuries), stress-related changes in blood physiology and post-release mortality in different reef-associated carcharhinid sharks (Skomal *et al*., 2007; Mandelman and Skomal, 2009). Most studies could relate overall fight time and/or environmental parameters, such as temperature, to changes in blood physiology, resulting in advice such as to avoid capture at high water temperatures (>31°C; Danylchuk *et al*., 2014). Additionally, Brooks *et al*. (2012) highlighted that using different fishing gear, for example long- compared with hand-lines or rod-and-reel angling, can potentially reduce the long-term physiological stress (based on the degree of acid–base disturbances) in captured Caribbean reef shark (*Carcharhinus perezi*). Indeed, recent studies on blacktip reef sharks in various injury scenarios have revealed that sharks heal quickly, even from severe physical wounds, prompting researchers to recommend that anglers catching sharks as bycatch should favour of cutting hooks or lines/leaders over holding sharks out of water or at the surface for prolonged periods of time while attempting to remove hooks (Chin *et al*., 2015). These conclusions have great potential for conservation practitioners that could adjust fishing gear guidelines (e.g. recommending circle hooks) accordingly. However, here and in many other physiological studies, concrete advice or suggestions for change of practices are often missing.

### Issue IV: ecotourism

Two recent reviews have collected studies investigating the effects of ecotourism and provisioning on sharks and rays, which are attractive and more sustainable alternatives to angling (Brena *et al*., 2015; Gallagher *et al*.; 2015). Most of their reviewed studies contributed information about how the controversial practice of provisioning affects diel activities, changes in movement and distribution patterns of sharks (few studies addressed the effects on rays) but rarely by using physiological approaches (see references in Brena *et al*., 2015). However, a single study on southern stingrays (*Dasyatis americana*) on the Cayman Islands used blood physiology to test the effects of wildlife tourism operations on individual stingrays (Semeniuk *et al*., 2009). In this study, the authors observed tourist-exposed stingrays not only to suffer from more injuries (e.g. boats strikes, bites from conspecifics) and higher parasite loads (potentially because of crowding conditions), but also stingrays exhibited decreased haematocrit and total serum proteins, indicating oxidative stress. In the following years, the authors did an exceptionally good job at integrating these blood physiology data with information on growth rates and survival of southern stingrays as well as tourist survey data into an integrated systems dynamics model that aimed at better management of tourist–stingray interactions (Semeniuk *et al*., 2010). By doing so, they efficiently translated their physiological results for the Cayman Island conservation practitioners and developed a profound way for a compromise between tourism and conservation claims which, not surprisingly, was highlighted as a success story of how to implement physiological findings for management and conservation (Madliger *et al*., 2016).

Very recently, Barnett *et al*. (2016) published another example of how physiology can contribute to an improved understanding and management of apex predators in tropical marine regions and gave precise management advice by measuring oxygen consumption rates of whitetip reef sharks (*Triaenodon obesus*) in the field. The authors found increased daily energy expenditures and could therefore recommend scheduling feeding operations with regard to frequency and timing (Barnett *et al*., 2016).

As expected, these aforementioned studies were able to link physiological findings to report thresholds for various species because the conservation problem they were investigating was limited in space, time and/or magnitude.

### Translating physiological findings for conservation use

Fish physiologists, including those researching coral reef fish species, are aware that they need to integrate their findings from molecular, cellular and, especially, individual levels to provide ecosystem-based management advice (Ward *et al*., 2016). Currently, approaches involving individual- or mass-based, as well as production, population or species distribution models are favoured because this information helps to inform conservation management more directly (Jørgensen *et al*., 2012; Metcalfe *et al*., 2012; Evans *et al*., 2015). Apart from the strong need for an interdisciplinary discourse between advice-delivering and advice-receiving parties who aim at a sustainable management and conservation of habitats and ecosystems, Jørgensen *et al*. (2012) highlighted that, in particular, the bioenergetics of predators and their prey will have to be considered to give appropriate management recommendations. In coral reef fishes, these types of studies are rather rare, but Cerino *et al*. (2013) have demonstrated how well physiology-based bioenergetics models can be used to establish a better understanding of the energy flow through ecosystems and to determine the potential impact, e.g. of invasive predators, such as the red lionfish (*Pterois volitans*) in the Caribbean Sea. Conservation efforts would benefit from site-specific and seasonal estimates of feeding rates, and clear advice could be given for removal strategies that reduce the pressure on local species or fisheries efforts. When investigating connectivity between coral reefs and the potential for coral reef fish recruitment, many elaborate biophysical and individual-based models have lately been equipped with vital information about developmental (pelagic larval duration; Fig. 1) and behavioural aspects of coral reef fish larvae, even using physiological data on swimming performance, but compared with the progress in modelling survival and growth of temperate fish species with physiological data, only sparse information exists on bioenergetics or the development of sensory abilities for coral reef fish larvae (Staaterman and Paris, 2014; Wolanski and Kingsford, 2014). Nevertheless, these types of models can provide valuable information, for example, on the placement and effectiveness of marine protected areas (e.g. cuban snapper; Kough *et al*., 2016). More information on individual growth, consumption rates and data on bioenergetics could provide valuable benefits toward improving these currently used models that help to translate physiological findings for conservation managers and contribute to a tangible and effective management and conservation of coral reef fishes.

### Implementation of physiological findings by conservation practitioners

Recent reviews from other systems show how physiological results can benefit management decisions, which may mean that some approaches can be adopted for the field of coral reef fish conservation physiology. For example, Madliger *et al*. (2016) have presented success stories from ‘eight areas of conservation concern, ranging from chemical contamination to invasive species to ecotourism, where physiological approaches have led to beneficial changes in human behaviour, management or policy’. What seemed particularly challenging was defining success, which can be species, site or system specific, but overall was defined as involving a change in human behaviour that is recommended because of physiological findings from the studies executed on the given topic and results in some level of conservation benefit. Although the team was optimistic, they too commented on the issues regarding accessibility of results and conveyed that most success stories came from piecing together data from multiple studies. Temperate examples of successes include improvements through physiological monitoring in fisheries management of various salmon species (see references in Madliger *et al*., 2016). As this team mentions conservation results are not always easily identified and may not even exist in the primary literature but rather government documents or websites and therefore disconnected from the original research, thus furthering the gap between researcher and management or conservation end point.

As described in the previous sections, the scientific recommendations to conservation managers in the primary literature can vary between vague general recommendations and very precise, temporally and spatially explicit instructions. In a recent review, Cvitanovic *et al*. (2015) pointed out that decision-makers still mostly rely on individual experience and knowledge rather than scientific evidence when deciding on specific management actions and highlighted the importance of knowledge exchange research for successfully implementing scientific findings into management decisions of marine resources. Other issues can come from cultural differences, institutional barriers and an overall lack of communication between researchers and end-users from the point of how research is designed through to how findings are translated (Cvitanovic *et al*., 2016). This is also true for coral reef fishes and ecosystems, but how can findings be better communicated to and implemented by conservation practitioners, given these challenges? Cooke and O'Connor (2010) have highlighted several ways of making conservation physiology relevant to policy-makers, mostly by improving communication, but also by training decision-makers in organismal biology to show them the full suite of options supporting their decision-making and to foster evidence-based conservation approaches. Before designing studies, stakeholders need to be identified; perhaps collaborators should be chosen, in part, communicate to specifically with stakeholders to co-develop research approaches and needs, and knowledge implementation needs to be monitored (e.g. Ningaloo Reef Marine Park; Cvitanovic *et al*., 2016). In addition, amassing support early on in the process, being able to deliver and showing success using a conservation physiology approach will help to assemble support for longer-term research programmes (Cooke *et al*., 2014).

### Outlook

Coral reef fishes face conservation problems today (e.g. bycatch for a specific fishery or habitat alterations) and require solutions for tomorrow. This includes answers to questions or issues that take decades to address, e.g. locations of marine protected areas and control of issues associated with climate change (see framework provided by Cooke *et al*., 2014). Although solutions for what are perceived as tomorrow's problems are required, those problems are also increasingly encroaching on coral reefs worldwide and becoming today's problems, as described earlier, which makes immediate solutions so difficult. Now and into the future, a strengthening of the interdisciplinary discourse and collaboration between coral reef fish physiologists, biologists and conservation practitioners will be needed for both identification of future research directions and successful implementation of findings (as indicated for other species and systems by Cooke and O'Connor, 2010; Horodysky *et al*., 2016). Providing summarized scientific information and communicating the value and applicability of findings to management practitioners will help physiological or evidence-based findings make it into the policy-making process and can lead to an overall better implementation of management decisions (Coristine *et al*., 2014; Walsh *et al*., 2015).

## Supplementary Material

Supplementary Data
